# Lokale Therapie von venösen Ulzera mit niedrig dosiertem rekombinantem, humanem „granulocyte-macrophage colony-stimulating factor“ (rhu GM-CSF), 10 Jahre Nachbeobachtung

**DOI:** 10.1007/s00105-022-05068-4

**Published:** 2022-10-26

**Authors:** Erna Jaschke, Julian Umlauft, Karin Palmer-Reichel, Wilhelm Oberaigner, Matthias Schmuth

**Affiliations:** 1 Innstr. 37, 6020 Innsbruck, Österreich; 2 Universitätsklinik für Dermatologie, Venerologie und Allergologie, Innsbruck, Österreich; 3 University for Health Sciences, Medical Informatics and Technology, Hall in Tirol, Österreich

**Keywords:** Chronische Wunden, Wundheilung, Humane Wachstumsfaktoren, Abheilraten, Rezidivraten, Chronic wounds, Wound healing, Human growth factors, Healing rates, Recurrence rates

## Abstract

**Hintergrund:**

Venöse Ulcera cruris treten in 1 % der Bevölkerung in industrialisierten Ländern auf. Ihre Behandlung ist schwierig und kostspielig. Eine komplette Abheilung zu erzielen ist langwierig, und die Rezidivrate ist hoch, bis zu 69 % im ersten Jahr nach der Abheilung.

**Fragestellung:**

In dieser Studie untersuchten wir, ob durch die lokale Therapie der venösen Ulzera mit niedrig dosiertem GM-CSF die Abheilraten verbessert und Rezidive verringert werden können.

**Methoden:**

Wir behandelten alle konsekutiven Patienten mit einem chronisch venösen Ulkus lokal mit einer niedrig dosierten GM-CSF-Lösung (10 µg/ml 0,9 %ige Kochsalzlösung, entsprechend einer Dosis von1,0–2,3 µg/cm^2^ Wundoberfläche). Alle Patienten waren über mehrere Wochen (median 8 Wochen) erfolglos mit anderen Lokaltherapien vorbehandelt. Bei allen Patienten erfolgte begleitend eine adäquate Therapie der venösen Insuffizienz.

**Ergebnisse:**

Von 130 Patienten sind bei 119 (91,5 %) Patienten die Ulzera komplett abgeheilt. Lokale oder systemische Nebenwirkungen wurden nicht beobachtet. Die mittlere Abheilzeit war 24 Wochen (median 14 Wochen). Wir konnten alle 119 abgeheilten Patienten nachverfolgen. Die mediane Nachbeobachtungszeit betrug 84 Monate. Die Rezidivrate betrug nach 1 Jahr 5,2 %, nach 4 Jahren 18,9 %, nach 10 Jahren 32,0 %

**Schlussfolgerung:**

Die Behandlung venöser Ulzera mit lokal appliziertem, niedrig dosiertem GM-CSF erwies sich als sichere, hochwirksame und kostengünstige Therapie. Die Abheilrate ist ebenso hoch wie in der ESCHAR Studie (Effects of Surgery and Compression on Healing And Recurrence in venous ulceration), und die Rezidivrate ist niedriger als in der Literatur angegeben. Die GM-CSF-Therapie erfolgte ausschließlich ambulant. Es waren keine Spitalaufenthalte notwendig.

## Abstract (en)

**Background:**

The treatment of leg ulcers is an enormous problem worldwide. Chronic venous ulceration affects 1% of the population and often has a protracted course. Recurrence rate is high, ranging up to 69% in the first year after healing.

**Objectives:**

To determine whether topical application of low-dose topical recombinant human granulocyte–macrophage colony-stimulating factor (rhu GM-CSF) is safe in venous leg ulcer treatment, and whether it accelerates healing rates and reduces recurrence rates.

**Materials and methods:**

Consecutive patients with chronic venous leg ulcers received topical treatment with low-dose rhu GM-CSF (10 µg/mL 0.9% sodium chloride solution; 1.0–2.3 µg rhu GM-CSF/cm^2^) in combination with treatment of venous insufficiency. All patients were previously treated with other topical wound remedies for several weeks (median 8 weeks) without success.

**Results:**

In 119 of 130 patients, the wounds healed completely (91.5%). No local or systemic adverse reactions were observed. The mean time to healing was 24 weeks (median 14 weeks). Median follow-up of the 119 patients with healed ulcers was 84 months. The recurrence rates were 5.2% after 1 year, 18.9% after 4 years and 32.0% after 10 years.

**Conclusions:**

Topical low-dose rhu GM-CSF proved to be safe and highly effective. Healing rates were comparable to those reported in the ESCHAR study (Effects of Surgery and Compression on Healing And Recurrence in venous ulceration) and recurrence rates were the lowest reported in the literature. Topical therapy with rhu GM-CSF can be applied in an outpatient setting and does not require hospitalization.

## Hintergrund und Fragestellung

Chronisch venöse Ulzera betreffen 1 % der erwachsenen Bevölkerung in industrialisierten Ländern. Die Abheilung dieser Geschwüre ist verzögert, und Rezidive sind häufig. Die dadurch entstehenden Kosten im Gesundheitssystem der entwickelten Länder sind hoch und betragen derzeit 1 % des Gesundheitsbudgets der Europäischen Union und auch anderer westlicher Staaten [2, 8, 19].

Der präzise Mechanismus des Auftretens von Ulzerationen bei chronischer venöser Insuffizienz ist noch nicht ganz geklärt. Die venöse Hypertension als Folge des venösen Refluxes, bedingt durch die Klappeninsuffizienz des oberflächlichen und/oder des tiefen Venensystems und der Perforansvenen, spielt eine wichtige Rolle [9, 19]. Es kommt zur Einwanderung von Neutrophilen, Makrophagen, Mastzellen und Lymphozyten, die an der Venenwand adhärieren und in die Venenwand und das umgebende Gewebe penetrieren. Multiple Chemokine, Zytokine, Proteasen und Matrixmetalloproteinasen werden produziert. Die Ablagerung von Kollagen Typ I und III ist dysreguliert. Es kommt zur persistierenden Entzündungsreaktion mit Störung der dermalen Bindegewebshomöostase und Ulkusbildung [21].

Die Kompressionstherapie ist der *Goldstandard* in der Behandlung der venösen Ulzera mit Abheilraten bis zu über 90 %. Allerdings rezidivieren die Ulzera trotz Kompressionstherapie häufig, 30–69 % bereits im ersten Jahr [2, 17, 19].

In der ersten Dekade dieses Jahrhunderts haben Barwell et al. und Gohel et al. in der ESCHAR-Studie (Effect of Surgery and Compression on Healing And Recurrence in venous ulceration) an einem Kollektiv von 500 Patienten zeigen können, dass durch chirurgische Sanierung des oberflächlichen Venensystems die Rezidive bei venösen Ulzera deutlich gesenkt werden können [2, 9]. Die Rezidivraten der Gruppe mit Kompression und Chirurgie lagen nach 4 Jahren bei 31 %, in der Gruppe der alleinigen Kompression bei 56 % [9]. Diese Ergebnisse wurden 2015 in einer Langzeitstudie über 10 Jahre bestätigt. Hier betrugen die Rezidivraten in der Gruppe Kompression und Chirurgie 48,9 %, in der Gruppe mit Kompression 94,3 % [8].

Die lokale Behandlung des oberflächlichen venösen Ulkus ist ebenso wichtig wie die Therapie der zugrunde liegenden venösen Hypertension. Die moderne, feuchte Wundbehandlung mit bioaktiven Wundverbänden kann die Heilung der venösen Ulzera unterstützen [24]. Aber es wurde bis heute nur teilweise herausgearbeitet, welche individualisierte Wundtherapie die beste für eine bestimmte Wunde ist [6, 20].

Große Erwartungen wurden in den Einsatz von Wachstumsfaktoren und Zytokinen zur Abheilung von chronischen Wunden gesetzt. Zytokine und Wachstumsfaktoren werden sowohl im Sekret akuter Wunden als auch in chronischen Wunden nachgewiesen. Im Wundsekret venöser Ulzera werden die Wachstumsfaktoren aber meist durch Proteinasen gespalten oder durch Makromoleküle abgefangen, sodass sie für die Wundheilung nicht zur Verfügung stehen [10, 18]. Wird ein Wachstumsfaktor als einzige Therapiemodalität eingesetzt, dann fördern die meisten Wachstumsfaktoren die Wundheilung nicht, möglicherweise, weil sie nur isoliert an bestimmten Stellen der Wundheilung eingreifen [12, 18].

GM-CSF ist nur zur Behandlung von Leukopenien nach Chemotherapien oder Knochenmarktransplantationen zugelassen. GM-CSF spielt als pluripotentes Zytokin eine zentrale Rolle im Wundheilprozess. GM-CSF wird bereits früh – rasch nach der Verwundung – durch Keratinozyten und Makrophagen produziert. Hämatogene Zellen (Granulozyten, Makrophagen, dendritische Zellen) und nichthämatogene Zellen (Keratinozyten, Fibroblasten, Endothelzellen) werden durch GM-CSF stimuliert. Es kommt in der Folge zu Neovaskularisation, Wundkontraktion und Reepithelisation. Zusätzlich hat GM-CSF auch einen Einfluss auf die Bildung der extrazellulären Matrix und die Kollagenproduktion. Im Mausmodell zeigen Tiere, die GM-CSF überexprimieren, eine verbesserte Wundheilung [13], während Tiere, die einen Antagonisten von GM-CSF überexprimieren, eine verschlechterte Wundheilung aufweisen [7, 14]. Im Jahr 1994 haben Robson et al. eine Vergleichsstudie an Ratten publiziert [22]. Die mit GM-CSF behandelten bakteriell infizierten Wunden zeigten eine schnellere Abheilung als jene, die nur mit dem Vehikel behandelt wurden.

Beim Menschen wurden im *Lancet* erstmals positive Effekte von GM-CSF auf die Heilung von Beinulzera berichtet [15]. Die darauf folgenden klinischen Fallserien und Studien wurden im Rahmen einer systematischen Übersichtsarbeit im Jahr 2011 zusammengefasst, wobei die Autoren die Wirksamkeit von GM-CSF für tiefe Brandwunden, Wunden bei Lepra und Beinulzera, nicht jedoch für Dekubitalulzera oder Wunden gesunder Individuen konstatierten [11].

Zuletzt haben wir unsere Ergebnisse mit topischer Applikation von GM-CSF an 38 Patienten mit 52 venösen Ulzera publiziert. Die Abheilrate betrug 90,4 % mit einer mittleren Abheilungsdauer von 19 Wochen, wobei auffiel, dass die Narbenbildung kosmetisch hervorragend war. In einer Nachbeobachtungszeit von 40 Monaten kam es lediglich bei 2 Patienten zum Auftreten von neuen Ulzera am selben Unterschenkel [12]. Im vorliegenden Manuskript berichten wir die Ergebnisse von 130 Patienten mit einer medianen Nachbeobachtungszeit von 84 Monaten.

## Studiendesign und Untersuchungsmethoden

### Patienten

In einem 10-Jahres-Zeitraum behandelten wir alle Patienten, die wegen eines vorbehandelten Unterschenkelgeschwüres die Ordination aufsuchten, lokal mit einer GM-CSF-Lösung. Von den 130 Patienten waren 101 Frauen und 29 Männer mit einem medianen Durchschnittsalter von 71 Jahren (Tab. 1).

**Table 1:** 

	Total	Gruppe 1 (GM-CSF-Spray)	Gruppe 2 (GM-CSF in Hydrofaser)
*Patientenanzahl*	130	43	87
Frauen	101	32	69
Männer	29	11	18
*Medianes Durchschnittsalter (Jahre)*	71 („range“ 24–97)	73 („range“ 27–94)	70 („range“ 24–97)
*Anzahl Patienten (%) mit:*			
„Superficial venous insufficiency“ (SVI)	87	21	66
„Postthrombotic syndrome“ (PTS)	41	21	20
Nicht definiert	2	1	1
*Mediane ***Ulkusgröße (cm**^**2**^**)**	9,0 („range“ 1–200)	9,0 („range“ 2–120)	9,0 („range“ 1–200)
*Medianer Vorbestand des Ulkus vor Therapiebeginn (Wochen)*	9,0 („range“ 0–1000)	8,5 („range“ 0–104)	9,0 („range“ 0–1000)

Die Patienten waren alle vorbehandelt mit verschiedensten Lokal- und Kompressionstherapien ohne wesentlichen Erfolg.

### Diagnostik und Therapie der chronisch venösen Insuffizienz

Die Diagnostik der chronisch venösen Insuffizienz erfolgte mittels klinischer Untersuchung, cw-Doppler-Ultraschall, Photoplethysmographie oder Phlebographie. Alle Patienten wurden klinisch als C6 der CEAP-Klassifikation klassifiziert [19]. Alle Patienten erhielten während der GM-CSF-Behandlung eine Kompressionstherapie mittels Kurzzugbandagen mit einem angestrebten Druck von 30 mm Hg (23–32 mm Hg). Eine operative Sanierung des oberflächlichen Venensystems nach Abheilung erfolgte nur bei Patienten, die jünger als 60 Jahre waren, insgesamt bei 9/119 Patienten (7,5 %, der Altersdurchschnitt unseres Gesamtkollektivs war 71 Jahre). Es erfolgte ein Venenstripping nach Babcock, d. h. Krossektomie (Unterbindung der V. saphena magna und der anderen einmündenden Venen in der Leiste) und anschließende Exhärese der insuffizienten Venen durch kleine Inzisionen sowie Unterbindungen der Perforansvenen.

### Ursprüngliche Ulkusgröße

Zu Beginn der Behandlung wurde die Ulkusgröße bestimmt durch die Messung der 2 maximalen horizontalen Durchmesser. Die mittlere Ulkusgröße betrug 20,6 cm^2^ (median 9,0 cm^2^); 73 Patienten wiesen eine Ulkusgröße kleiner als 10 cm^2^ auf, 55 Patienten eine Fläche größer als 10 cm^2^, bei 2 Patienten wurde die Ulkusgröße nicht bestimmt.

### Laboruntersuchungen

Bei allen Patienten wurden vor der Therapie Blutsenkung, Differenzialblutbild, Blutzucker, hepatische und renale Funktionen erfasst. Diese Tests wurden im Laufe der Therapie regelmäßig kontrolliert, üblicherweise 1‑mal im Monat.

### Allergologische Untersuchungen

Bei 111 der 130 Patienten wurde ein Epikutantest der Standardreihen, der Salbengrundlagen und Konservierungsmittel (Almirall Hermal, Reinbek, Deutschland) durchgeführt. Auch die verwendeten Wundauflagen und die GM-CSF-Lösung wurden epikutan getestet.

### Bakteriologische Untersuchungen

Bei klinischen Zeichen einer Infektion (Rubor und Calor im Bereich der Wundumgebung) wurde bei 82/130 Patienten (63 %) ein mechanisches Wunddébridement durchgeführt und ein Abstrich oder Material aus den tiefen Wundabschnitten entnommen.

### Dosierung GM-CSF

Robson et al. zeigten im Tierversuch an Ratten, dass bakteriell infizierte Wunden unter Lokaltherapie mit rhu GM-CSF signifikant schneller abheilten als die Kontrollen, wobei 1 µg/cm^2^ Wundoberfläche ebenso wirksam war wie 10 µg/cm^2^ Wundoberfläche [22].

Um diese Dosierung auf der Oberfläche der venösen Ulzera zu erreichen, haben wir bei den Patienten Nr. 1–43 (Gruppe 1) den kommerziell erhältlichen rhu GM-CSF (Leucomax®, Novartis, Aesca, Österreich) in einer physiologischen Kochsalzlösung in einer Konzentration von 10 µg/ml aufgelöst und auf die Wunden aufgesprüht. Eine 1‑ml-Lösung ist ausreichend, um 10 cm^2^ Wundoberfläche zu bedecken, sodass eine Konzentration von 1,0 µg/cm^2^ besprühter Oberfläche erreicht wurde. Anschließend wurde ein Sekundärverband mit einer nichtadhärenten Wundkompresse (Vliwin®, Lohmann & Rauscher, Deutschland) aufgebracht. Bei den Patienten Nr. 44–130 (Gruppe 2) inkorporierten wir diese GM-CSF-Lösung in eine Hydrofaser (Aquacel Extra, ConvaTec Limited, Deeside, Flintshire, UK). Die Hydrofaser wandelte sich dabei in ein Gel um. Die Dosierung, die durch diese Art der Applikation auf der Wundoberfläche erreicht wurde, entsprach 2,3 µg GM-CSF/cm^2^ in der Hydrofaser. Wir haben diese Applikationsform gewählt, da sie leichter und sicherer anzuwenden ist als der GM-CSF-Spray, v. a. von medizinischem Hilfspersonal, das die Verbandwechsel 2‑mal wöchentlich zwischen den Ordinations- bzw.- Hausbesuchen (üblicherweise alle 14 Tage) durchführte. Bei allen Patienten erfolgte eine adäquate, konservative, venöse Kompressionstherapie [12].

### Langzeituntersuchungen

Klinische Verlaufsuntersuchungen aller noch lebenden Patienten wurden mit einer medianen Nachbeobachtungszeit von 84 Monaten durchgeführt. Wenn die Patienten in der Zwischenzeit verstorben waren, suchten wir Kontakt zu den Angehörigen, Hausärzten, Altersheimen oder Spitälern, um den Endzustand der abgeheilten Wunden und die Todesursache zu eruieren.

### Statistische Daten

Für die Beschreibung der Patientencharakteristik wurden relative Häufigkeiten berechnet und angegeben. Abheilrate und Rezidivrate wurden mit der Kaplan-Meier-Methode geschätzt.

Für die Berechnung der Abheilrate wurde die Zeit von Beginn der Behandlung bis entweder Heilung oder Therapieende analysiert. Als Event wurden alle Abheilungen gezählt. Wir haben nur komplett abgeheilte Patienten analysiert.

Für die Berechnung der Rezidivrate der geheilten Patienten (*n* = 119) wurde die Zeit von der Abheilung bis zum Rezidiv oder Tod oder Ende der Beobachtung analysiert, als Event wurde ein Rezidiv gezählt.

Die statistische Analyse wurde mit Stata Version 17 durchgeführt.

## Ergebnisse

### Diagnostik der chronisch venösen Insuffizienz

Alle Patienten wurden klinisch als C6 der CEAP-Klassifikation klassifiziert [19]. Von 130 Patienten wurde bei 87 Patienten eine superfizielle chronische venöse Insuffizienz (SVI) diagnostiziert. Bei 41 Patienten bestand ein postthrombotisches Syndrom (PTS *=* tiefe Leitveneninsuffizienz). Bei 2 dieser Patienten wurde diese nicht bestimmt (Tab. 1).

### Abheilrate, Abheildauer und Nebenwirkungen

**Figure 1:**
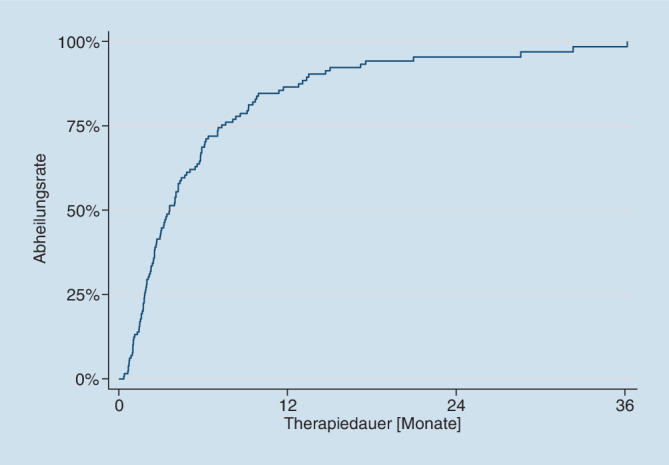

Von den 130 behandelten Patienten sind 119 (*91,5* *%*) komplett abgeheilt (Tab. 2, Abb. 1). Die mittlere Abheildauer für alle Patienten betrug 24 Wochen (median 14 Wochen). Die Abheilrate war schlechter bei Patienten mit Ulzera, die größer als 10 cm^2^ waren, bei Patienten mit PTS und bei Patienten mit Vorbestand länger als 26 Wochen. Lokale und systemische Nebenwirkungen konnten nicht beobachtet werden. Die Lokalbehandlung mit GM-CSF war schmerzfrei.

**Table 2:** 

	Total	Gruppe 1	Gruppe 2
*Insgesamt*	119 (91,5 %)	40 (93,0 %)	79 (90,8 %)
Frauen	92 (93,1 %)	29 (90,6 %)	63 (91,3 %)
Männer	27 (91,1 %)	11 (100 %)	16 (88,9 %)
*Anzahl Patienten mit:*			
„Superficial venous insufficiency“ (SVI)	83 (95,4 %)	21 (100 %)	62 (93,3 %)
„Postthrombotic syndrome“ (PTS)	35 (85,4 %)	19 (90,5 %)	16 (80,0 %)
Nicht definiert	1	–	1
*Mediane ***Ulkusgröße (cm)**	9,0 („range“ 1–200)	9,0 („range“ 2–120)	9,0 („range“ 1–200)
*Mediane Abheildauer (Wochen)*	14 („range“ 1,4–157,3)	14 („range“ 1,7–50,1)	14 („range“ 1,4–157,3)
*Medianer Vorbestand des Ulkus vor Therapiebeginn (Wochen)*	9,0 („range“ 0–1000)	8,5 („range“ 0–104)	9,0 („range“ 0–1000)

### Labortestungen

Die regelmäßig durchführten Laborparameter zeigten keine wesentlichen Veränderungen gegenüber den Ausgangsbefunden. Insbesondere konnte keine Leukozytose nachgewiesen werden.

### Allergologische Tests

Um zu verhindern, dass durch Sensibilisierungen ein Bias für die Abheilung entsteht, wurden 111/130 Patienten einer Epikutantestung unterzogen. Die Rate positiver Testungen entsprach der publizierten Rate von Kontaktsensibilisierungen bei Ulkuspatienten. Die häufigsten positiv getesteten Allergene in unserem Kollektiv waren Wollwachsalkohole, gefolgt von Perubalsam und Neomycin.

Bei positivem Epikutantest wurde eine strikte Allergenkarenz auf die jeweilige Substanz durchgeführt. Keiner der 111 getesteten Patienten reagierte im Epikutantest auf die GM-CSF-Lösung positiv.

### Bakteriologische Untersuchungen

Es zeigten 82/130 Patienten *(63* *%)* vorübergehend klinisch Zeichen einer Infektion. Das Keimspektrum wurde mittels Abstrich vom Wundgrund bestimmt.

Patienten mit Rubor und Calor im Bereich der Wundumgebung wurden systemisch mit Antibiotika entsprechend dem Antibiogramm behandelt.

### Unterschiede zwischen GM-CSF-Spray (Gruppe 1) versus GM-CSF in Hydrofaser (Gruppe 2)

Die Abheilraten in den beiden Gruppen waren vergleichbar. Die mediane Abheildauer betrug in beiden Gruppen 14 Wochen (Tab. 2). Die Rezidivraten der Patienten in der Gruppe 2 waren etwas niedriger als in der Gruppe 1.

### Langzeitergebnisse

**Figure 2:**
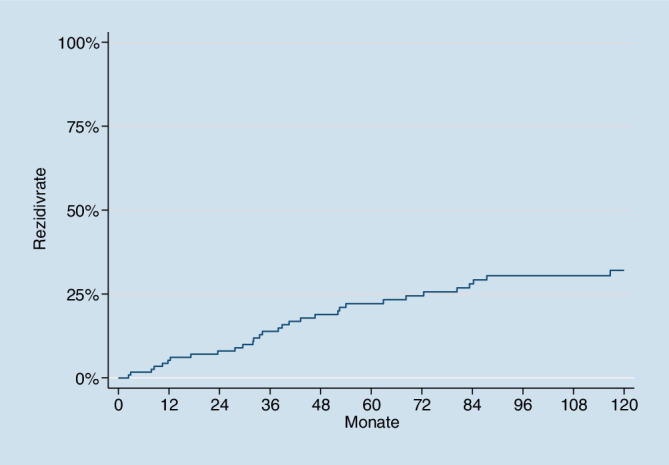

Wir konnten alle 119 abgeheilten Patienten nachverfolgen mit einer medianen Nachbeobachtungszeit von 84 Monaten. Dass wir alle abgeheilten Patienten nachverfolgen konnten, klingt ungewöhnlich und auch unwahrscheinlich. Wir führen das auf die besondere geografische Situation – beschränkt auf einen politischen Bezirk –, auf die eingeschränkte Mobilität der älteren Bevölkerung und auf die guten Kontakte zu Angehörigen, Hausärzten, Krankenanstalten und Altersheimen zurück. Es war mühsam, aber auch befriedigend, da wir keine ablehnenden, sondern eher positive Reaktionen erleben konnten. Rezidive traten nach 1 Jahr in 5,2 % der Patienten auf, nach 2 Jahren in 8,0 %; nach 3 Jahren in 13,9 %, nach 4 Jahren in 18,9 % und nach 10 Jahren in 32,0 % (Tab. 3, Abb. 2). Die abgeheilten Wunden waren stabil und kosmetisch hervorragend akzeptabel. Atrophe oder hypertrophe Narben wurden nicht beobachtet (sind allerdings bei venösen Ulzera im Gegensatz zu Brandwunden ohnehin selten).

**Table 3:** 

Rezidiv (Jahre nach Abheilung)	Marston *N* = 202	Gohel ESCHAR-Studie *N* = 500		Van Gent *N* = 80		Jaschke *N* = 119
	Kompression (%)	Kompression (%)	Kompression + Chirurgie (%)	Kompression (%)	Kompression + Chirurgie (%)	Kompression + Gm‑csf (%)
1	21	–	–	–	–	5,2
2	29	–	–	–	–	8,0
3	38	–	–	–	–	13,91
4	–	56	31	–	–	18,9
10	–	–	–	94,3	48,9	32,0

## Diskussion

Dies ist eine prospektive Kohortenstudie der topischen Behandlung venöser Ulzera mit rhu GM-CSF. Zuvor hatten wir unsere Ergebnisse an 38 Patienten mit 52 Ulzera publiziert. Die Abheilrate betrug 90,4 % mit einer mittleren Abheilungsdauer von 19 Wochen. In einer Nachbeobachtungszeit von 40 Monaten kam es lediglich zum Auftreten von 2 neuen Ulzera am selben Unterschenkel bei 2 Patienten [12].

In dem jetzt vorgestellten Kollektiv mit 130 Patienten war die Abheilrate *91,5* % mit einer mittleren Abheildauer von 24 Wochen (median 14 Wochen). Wir konnten 119 abgeheilte Patienten nachverfolgen. Rezidive wurden nach 10 Jahren bei nur 32,0 % der Patienten beobachtet (Tab. 3, Abb. 2).

Die beschriebenen Ergebnisse mit lokaler GM-CSF-Therapie kombiniert mit Kompression ohne chirurgische Intervention sind besser als die in der Literatur angegebenen Rezidivraten nach chirurgischer Intervention und Kompression (Tab. 3). Vergleichszahlen unter venöser Kompressionstherapie stammen von Marston et al. [17]. Es wurden 252 Patienten mit venösen Ulzera mit Kompression behandelt; 96 % der Ulzera heilten innerhalb eines Jahres komplett ab; 202 Patienten wurden nachkontrolliert. Die Rezidivrate betrug nach 1 Jahr 21 %, nach 2 Jahren 29 %, nach 3 Jahren 38 %. Im Jahr 2007 publizierten Gohel et al. die ESCHAR-Studie mit 500 Patienten, davon 258 in der Gruppe mit Kompression und Chirurgie und 242 in der Kompressionsgruppe. Die Abheilraten betrugen 89 % in der Kompressionsgruppe und 93 % in der Gruppe mit Kompression und Chirurgie [9]. Die Rezidivrate betrug nach 4 Jahren 56 % vs. 31 % [9]. Im Jahr 2015 wurden diese Ergebnisse in einer Langzeitstudie von 10 Jahren bestätigt. Die Rezidivrate nach 10 Jahren Nachbeobachtung betrug für die Chirurgie- und Kompressionsgruppe 48,9 % und für die Kompressionsgruppe 94,3 % [8].

Unter der topischen Anwendung von GM-CSF sind in der vorliegenden Kohorte keine lokalen oder systemischen Nebenwirkungen beobachtet worden. Im Gegensatz zur topischen Applikation eines anderen Wachstumsfaktors, „platelet-derived growth factor“ (PDGF), für den es einen Rote-Hand-Brief gibt bezüglich einer möglichen Tumorneogenese, ist für GM-CSF auch in systemischer Anwendung (Therapie der Neutropenie nach Knochenmarktransplantation und Chemotherapie) keine Assoziation mit sekundären Malignomen bekannt.

Es ist davon auszugehen, dass die Applikationsform von GM-CSF die Effektivität beeinflusst. In mehreren publizierten Studien wurde GM-CSF subkutan periläsional um die Wunden herum injiziert. Diese Art von intraläsionaler Applikationstherapie kann systemische Nebenwirkungen wie Kollaps, Lumbalgien, abdominale Beschwerden und Übelkeit verursachen, wie sie auch für die Anwendung des GM-CSF für hämatologische Indikationen bekannt sind. Diese Nebenwirkungen werden üblicherweise als moderat beschrieben [1, 3, 5, 15, 16]. Wir haben eine Sprayapplikation mit der Applikation einer GM-CSF-getränkten Hydrofaser verglichen und keinen signifikanten Unterschied feststellen können. Die Annahme, dass die Dosis von 2,3 µg GM-CSF/cm^2^ zu einer höheren Abheilrate führen könnte (Hydrofaser), hat sich nicht bestätigt. Das könnte darauf zurückzuführen sein, dass die Konzentration von 2,3 µg GM-CSF/cm^2^ nur in der Hydrofaser, nicht aber auf der Wundoberfläche erreicht wurde oder dass – wie von Robson beschrieben – durch eine höhere Dosis als 1 µg/cm^2^ Wundoberfläche keine wesentliche Besserung in der Abheilrate erreicht werden kann. Um die Bioverfügbarkeit von GM-CSF in der Wunde weiter zu verbessern, wäre eine Injektion von GM-CSF produzierenden Zellen in Erwägung zu ziehen [23].

Studien aus China berichten von der topischen Applikation von GM-CSF-Gel bei tiefen Verbrennungswunden. Die Abheilzeiten waren in den GM-CSF-Gruppen kürzer als in den Kontrollen, die nur mit Vehikel behandelt wurden. Außerdem traten in den mit GM-CSF behandelten Arealen weniger Infektionen auf. Lokale oder systemische Nebenwirkungen wurden nicht nachgewiesen [4, 11, 25–28].

Leider fehlen in den publizierten Studien Langzeitbeobachtungen. Diese Lücke schließt unsere gegenwärtige Auswertung mit einer medianen Nachbeobachtungszeit von 84 Monaten. Auffällig waren in unserem Kollektiv die kosmetisch hervorragenden und stabilen Narben. Für die Behandlung von Brandwunden könnte die lokale Therapie mit GM-CSF eine überschießende Narbenbildung mit hypertrophen Narben und Strikturen verhindern.

Limitierend ist, dass unsere Studie nicht den Erfordernissen einer randomisierten kontrollierten Studie entspricht und dass keine Daten zum Vorliegen eines arthrogenen Stauungssyndroms oder einer Adipositas-assoziierten chronischen Veneninsuffizienz erhoben wurden. Dies ist insbesondere beim Vergleich der beiden Gruppen zu berücksichtigen. Durch die aufwendige Nachuntersuchung aller abgeheilten Patienten konnten wir jedoch feststellen, dass viele Patienten Jahre bis Jahrzehnte lang ohne Ulkus überlebten oder ulkusfrei starben. Wir glauben, dass die exakte Dokumentation und Nachbeobachtung die reelle Situation in der klinischen Praxis reflektiert. Wir haben alle konsekutiven Patienten mit chronisch venösen Ulzera therapiert und analysiert. Dies sollte Anlass für weitere Untersuchungen in diese Richtung sein.

Ein weiterer limitierender Faktor sind Kosten. Da die GM-CSF-Therapie ausschließlich ambulant durchgeführt wurde, keine stationären Behandlungen notwendig waren und die Patienten nach abgeschlossener Therapie jahrelang rezidivfrei waren, kann jedoch der Gesamtverlauf als kostengünstig angesehen werden.

## Fazit für die Praxis

- GM-CSF-Behandlung von chronischen Ulzera (venöse Ulzera, tiefe Verbrennungen) fördert die Granulation und die Reepithelisierung.
- Nach GM-CSF-Behandlung von venösen Ulzera sind die Abheilraten hoch und die Rezidivraten niedrig.
- GM-CSF-Behandlung chronischer Wunden führt zu kosmetisch akzeptabler Narbenbildung.
